# 21. IDENTIFYING INDIVIDUALS AT HIGH RISK FOR SCHIZOPHRENIA: LJ SEIDMAN MEMORIAL SYMPOSIUM

**DOI:** 10.1093/schbul/sby014.084

**Published:** 2018-04-01

**Authors:** Lynn DeLisi

**Affiliations:** VA Boston Healthcare System, Brockton Division

## Abstract

**Overall Abstract:** Lawrence J. Seidman was born and grew up in New York City. He obtained a PhD from Boston University in Psychology and stayed in Massachusetts to work for many years in the Harvard affiliated Hospitals, such as the VA-Boston Healthcare System, Massachusetts General Hospital and at his untimely passing, he was Professor of Psychology in the Department of Psychiatry at Beth Israel-Deaconess Hospital and the Massachusetts Mental Health Center. He was about to move to a new phase in his career, assuming a position at Children’s Hospital, Boston. He was a pioneer in the fields of the neuropsychology of schizophrenia, ADHD and related disorders, of using the tools of cognitive assessments and brain imaging to understand the genetic predisposition for serious mental illness, and in the last several years—prediction of conversion to psychosis in individuals at high risk. He contributed to many multicenter collaborations and had several collaborators world-wide, playing an important role in their work. This symposium is conducted in his honor with contributions from key collaborators on different aspects of his work. Drs. Tyronne Cannon and Elaine Walker will both represent the North American Prodrome Longitudinal Study (NAPLS) consortium by reviewing its findings in brain imaging and cognition. Dr. David Braff will review the work of the Consortium on the Genetics of Schizophrenia (COGS) multicenter collaboration, in which Dr. Seidman led one of its sites, and Dr. TianHong Zhang from Shanghai will present current data from the Shanghai-Boston SHARP collaboration on early detection of psychosis. The symposium will be concluded by Dr. Keshavan from Beth Israel-Deaconess, who was a close colleague of Dr. Seidman for the last decade. Together they worked side by side exploring many aspects of early detection of schizophrenia and educating trainees and the public on their findings. He will sum up the legacy of Dr. Seidman to the field and how we can continue it into the future.

